# Insecticide resistance and underlying targets-site and metabolic mechanisms in *Aedes aegypti* and *Aedes albopictus* from Lahore, Pakistan

**DOI:** 10.1038/s41598-021-83465-w

**Published:** 2021-02-25

**Authors:** Rafi Ur Rahman, Barbara Souza, Iftikhar Uddin, Luana Carrara, Luiz Paulo Brito, Monique Melo Costa, Muhammad Asif Mahmood, Sozaina Khan, Jose Bento Pereira Lima, Ademir Jesus Martins

**Affiliations:** 1 Laboratório de Fisiologia E Controle de Artrópodes Vetores, Instituto Oswaldo Cruz/FIOCRUZ, Av. Brasil 4365, Rio de Janeiro, RJ 21040-360 Brazil; 2 Community Medicine Department, Bacha Khan Medical College Mardan, Mardan, Pakistan; 3 Institute of Public Health, Lahore, Pakistan; 4 Zoology Department, Government College University, Lahore, Pakistan; 5 Instituto Nacional de Ciência e Tecnologia em Entomologia Molecular, INCT-EM, UFRJ, Rio de Janeiro, RJ 21941-902 Brazil

**Keywords:** Biological models, Gene expression analysis, Genetic techniques, Viral infection

## Abstract

Insecticide resistant *Aedes* populations have recently been reported in Pakistan, imposing a threat to their control. We aimed to evaluate the susceptibility of *Aedes aegypti* and *Aedes albopictus* populations from Lahore to WHO-recommended insecticides and to investigate metabolic and target-site resistance mechanisms. For this purpose, we first carried out bioassays with the larvicides temephos and pyriproxyfen, and the adulticides malathion, permethrin, deltamethrin, alpha-cypermethrin, and etofenprox. We looked for Knockdown resistance mutations (*kdr*) by qPCR, High-Resolution Melt (HRM), and sequencing. In order to explore the role of detoxifying enzymes in resistance, we carried out synergist bioassay with both species and then checked the expression of *CYP9M6*, *CYP9J10*, *CYP9J28, CYP6BB2, CCAe3a*, and *SAP2* genes in *Ae. aegypti*. Both species were susceptible to organophosphates and the insect growth regulator, however resistant to all pyrethroids. We are reporting the *kdr* haplotypes 1520Ile + 1534Cys and T1520 + 1534Cys in high frequencies in *Ae. aegypti* while *Ae. albopictus* only exhibited the alteration L882M. PBO increased the sensitivity to permethrin in *Ae. aegypti*, suggesting the participation of P450 genes in conferring resistance, and indeed, *CYP928* was highly expressed. We presume that dengue vectors in Lahore city are resistant to pyrethroids, probably due to multiple mechanisms, such as *kdr* mutations and P450 overexpression.

## Introduction

Lahore, the second-largest Pakistani city, also known as Pakistan’s cultural capital, has faced several outbreaks of dengue in the last two decades. The biggest of which was in 2011 when more than 20,000 people were hospitalized and 350 died^1^. Dengue virus has four serotypes, and all four have been reported in Lahore^2^. However, serotype 2 (genotype IV) was more prevalent and was responsible for high mortality and morbidity in Lahore^3^. The most recent outbreaks in the country took place in 2019 when more than 47,000 patients were admitted to hospitals (up to November) due to mild dengue fever, severe hemorrhagic fever, or shock. Lahore was once again the hotspot of the infection^4^.

Dengue virus is transmitted to humans by the bite of a female infected *Aedes aegypti* Linnaeus, 1762 and *Aedes albopictus* Skuse, 1895 (Diptera: Culicidae). The recent spread of the vector population and global environmental conditions, such as the average temperature increase, has promoted the disease's transmission beyond the tropics^5,6^. In the absence of a preventive vaccine and potent treatment, effective vector management remains the best option to keep dengue under control^7^. Both *Aedes* species are abundant in Pakistan, with *Ae. albopictus* found at high altitude and in peri-urban areas and *Ae. aegypti* most abundant in urban settlements. In Lahore, both mosquito species are found, but *Ae. aegypti* is relatively more abundant^8^. Apart from dengue, chikungunya was detected in patients from Karachi and Lahore^9,10^. So far, there is no evidence of Zika and yellow fever transmission in Pakistan.

Since the beginning of the dengue outbreaks in Lahore, neurotoxic insecticides like pyrethroids (PY), mainly permethrin and deltamethrin, have been used as adulticides, while the organophosphate (OP) temephos as a larvicide. When a case of dengue is reported, comprehensive vector surveillance and health education activities are conducted in all houses at a radius of approximately 200 m from the index house. Potential breeding sites are searched and mechanically eliminated, temephos is applied in containers that cannot be managed physically. Alpha-cypermethrin (10% SC) is applied as a residual spray on the expected vector's resting sites. All houses or industrial areas present in a 50 m radius are also sprayed with this pyrethroid. Deltamethrin was used for vector control before the introduction of alpha-cypermethrin. In addition to case response, larvicide temephos is used during routine vector surveillance in addition to the mechanical elimination of potential vector breeding sites in the Punjab province. The option of space spray is used during outbreaks only under the advice of a technical committee. All vector control practices, including space spraying and larvicides applications, are applied by the public sector. Community is well educated to eliminate possible breeding places from their houses and workplaces mechanically. Legal action is taken against the occupants of the premises in non-compliance with the instructions regarding eliminating mosquito breeding sites in their premises^11^. Resistance against pyrethroids in *Aedes* has been reported from various parts of Punjab^12^ and Khyber Pakhtunkhwa provinces^13^. Similarly, in the Indian capital city of New Delhi, which is only 425 km from Lahore, pyrethroid-resistant *Ae. aegypti* are prevalent^14^. Resistance to temephos in *Aedes* has also been shown in several districts of Punjab province, including Lahore and its neighbor districts^15^. On the other side, Insect Growth Regulators (IGRs) were effective against *Aedes* in the area, at least until 2008^16^.

Neurotoxic insecticides interfere with the central nervous system of insects. Pyrethroids (PYs) and the organochlorines (OCs) target the voltage-sensitive sodium channel (*Na*_*V*_, also commonly referred to as *Vssc* or *Vgsc*). This protein comprises four homologous domains (I-IV), each containing six transmembrane subunits of α-helix (S1–S6) and a P-loop connecting S5 and S6 segments. The first four segments make the voltage-sensing domain (S1–S4), while the last two segments (S5 and S6) along with their respective P-loops assemble in such a way to form the pore, which is responsible for the action potential of ions, particularly Na^+^. Upon binding, PY and OC keep this pore or channel open for more extended periods resulting in fast contractions, paralysis, and eventual death, known as the *knockdown* effect^17–19^. Pyrethroids are either type I or type II based on their chemical structure. Type I (for example, permethrin) lack an alpha-cyano group, while type II (for example, deltamethrin and cypermethrin) have an alpha-cyano group close to phenyl benzyl alcohol moiety. On the other hand, organophosphates (OPs) and carbamates (CAs) target the acetylcholinesterase (AChE, EC 3.1.1.7) coded by the *ace-1* gene^18^. Insect Growth Regulators (IGRs) mimic the growth hormones, not directly killing the insect but affecting their growth, development, physiology, and behavior, ultimately provoking the insect death^20^.

Continuous application of insecticides results in the selection of resistant insects. Resistance mechanisms in arthropod vectors are provoked by behavioral or physiological changes. Physiological responses in the form of target-site modifications alone can be sufficient to confer pyrethroids resistance in *Aedes*. One or more point mutations can achieve resistance to pyrethroids' knockdown effect (*kdr*) in the voltage-gated sodium channel gene (*Na*_*v*_). About 11 *kdr* mutations alone or in combinations are classically associated with PY and DDT resistance in *Aedes*. Of them, Val410Leu (IS6 segment), Ser989Pro (IIS6 segment), Val1016Ile/Gly (IIS6 segment), and Phe1534Cys (IIIS6 segment) are well studied^21^. A more recently described mutation, Thr1520Ile (IIIS6 segment), is also associated with PY resistance in *Ae. aegypti* populations from India^22^ and Thailand^23^. The 1534Cys is the only *kdr* mutation widespread on a global scale. On the other hand, mutation at the 1016 site varies geographically, as Val1016Gly is predominant in populations from Asia while Val1016Ile occurs in Latin American^23^. Surprisingly, Val1016Ile has also been detected in Vietnam as well^24^, and Val1016Gly in Panama^25^. Of other notable mutations, Val410Ile is restricted to Latin America and Ser989Pro to Asia, but they alone do not cause resistance^21^. The 1534C alone can confer resistance to PYs, and this resistance can reach higher levels when combined with other mutations like 989P, 1016Ile/Gly, or 1520I^26^. Contrary to pyrethroids, target site modification in OP resistance, Gly119Ser (G119S) in the *ace-1* gene of other mosquito species is not likely to occur in the genus *Aedes* due to codon constraints^7,27^.

Metabolic resistance mechanisms are also very well investigated in *Ae. aegypti*, aiding in xenobiotic detoxification through enhanced expression or gene alterations^28^. Enzymes that detoxify neurotoxins are mainly from the cytochrome P450 dependent multi-function oxidases (CYP450 MFOs), esterases (ESTs), or glutathione-S-transferases (GSTs) families^28^. Around 160 MFOs^29^ have been reported to have altered expression in the resistant *Aedes* population, of which *CYP9J22, CYP9J26, CYP9J28*, *CYP9M6*, *CYP9M9, CYP6BB2*, and *CYP9J10* have been strongly associated with PY resistance. The esterase gene *CCEAe3a* is generally overexpressed in OP resistant populations^7,30^. A recent observation was the overexpression of a sensory appendage protein (*SAP2*) in PY resistant *Anopheles gambiae* population from Burkina Faso^31^.

In this study, we have evaluated the susceptibility/resistance profile of *Ae. aegypti* and *Ae. albopictus* populations from Lahore to the insecticides in practice and the alternative compounds IGR pyriproxyfen. We investigated the main molecular mechanisms involved in insecticide resistance in *Aedes* populations from Lahore for the first time. These results will enrich the knowledge about insecticide resistance in Pakistani *Aedes* species and contribute to vector control monitoring actions that would ultimately reduce Pakistan's arboviruses burden.

## Results

### Bioassays with larvicides

#### Temephos

**Figure 1:**
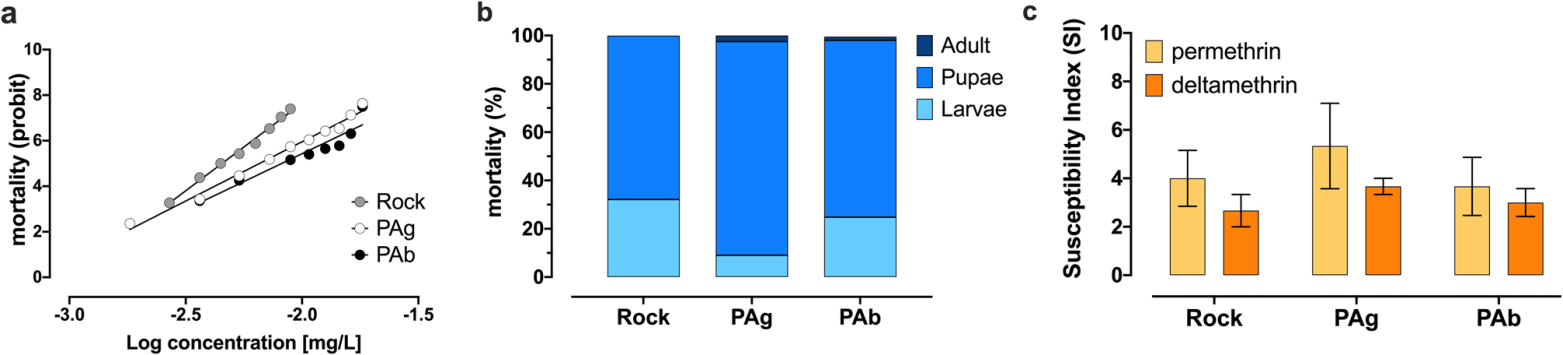
Larvicide bioassays with *Aedes aegypti* and *Aedes albopictus* from Lahore, Pakistan. (**a**) Dose–response bioassay to the organophosphate temephos, with mortality evaluated after 24 h of exposure. (**b**) Proportional mortality of each life stage to the diagnostic dose of the IGR pyriproxyfen. The adult emergence inhibition (AEI) was 100% in all experimental cases. (**c**) Susceptibility index (SI) average (with SEM error bars) for the pyrethroids permethrin and deltamethrin. The higher the SI, the less susceptible the population. *Ae. aegypti* Rockefeller strain (Rock) was used as a reference in all these larvicide bioassays.

Dose–response assays determined the lethal concentrations (LCs) and resistant ratios (RRs) in *Aedes aegypti* (PAg) and *Aedes albopictus* (PAb) populations from Lahore, Pakistan, with serial dilutions from 0.001 to 0.018 mg/L (Fig. 1a). The LC_50_ for *Aedes aegypti* (PAg) and *Aedes albopictus* (PAb) populations were 0.006 mg/L and 0.008 mg/L, with respective RR_50_ as 1.50 and 2.04, employing *Ae. aegypti* Rock as a baseline reference. Accordingly, LC_90_ were 0.011 and 0.014, with RR_90_ of 2.0 and 2.3 for PAg and PAb, respectively (Table 1).

**Table 1:** Susceptibility profile to the larvicide organophosphate temephos in *Aedes aegypti* and *Aedes albopictus* from Lahore, Pakistan.

Population	LC50 (CI95%)	LC90 (CI95%)	RR_50_^a^	RR_90_^a^	Slope
*Ae. aegypti* (PAg)	0.006 (0.004–0.009)	0.011 (0.007–0.016)	1.5	1.8	5.1
*Ae. Albopictus* (PAb)	0.008 (0.003–0.018)	0.014 (0.005–0.039)	2.0	2.3	4.9
*Ae. aegypti* (Rock)	0.004 (0.003–0.006)	0.006 (0.005–0.008)	**–**	**–**	

^a^ Resistance ratios (RR) were calculated based on *Ae. aegypti* Rockefeller.

#### Pyriproxyfen

We performed a qualitative bioassay (diagnostic dose assay) with the insect growth regulator (IGR) pyriproxyfen using a diagnostic dose of 0.3 µg/L. There was no mortality in the control condition, which generally lasted for 7 to 10 days until all pupae emerged into adults, indicating completion of tests. At this point, mortality ranged from 99.4% to 100% in the experimental condition, and the higher proportions of mortality occurred in the pupal stage. Rock exhibited the highest proportion of mortality in the larval stage (32.2%), compared to PAg (9.03%) and PAb (24.8%). Pyriproxyfen induced 100% of Adult Emergence Inhibition (AEI) in the reference lineage and in both PAg and PAb populations (Fig. 1b).

#### Pyrethroids (rapid knockdown assay)

For Rock, PAg and PAb, there was 100% knockdown in 0.1 ppm and 0.4 ppm for permethrin and deltamethrin. In total, we exposed 60 larvae of each strain to both pyrethroids. The susceptibility index (SI) average for deltamethrin was 2.7 in Rock, 3.0 in PAb and 3.7 in PAg, and for permethrin, it was 4.0 in Rock, 3.7 in PAb, and 5.3 in PAg (Fig. 1c). Although this data roughly suggested a higher SI to PAg for both pyrethroids, the difference among the strains was not significant, according to the ANOVA test: deltamethrin (P = 0.4640) and permethrin (P = 0.6892).

### Bioassays with adulticides

#### Malathion

Both PAg and PAb exhibited 100% mortality showing susceptibility to the organophosphate malathion (20 μg/mL), similar to Rock.

#### Pyrethroids

**Figure 2:**
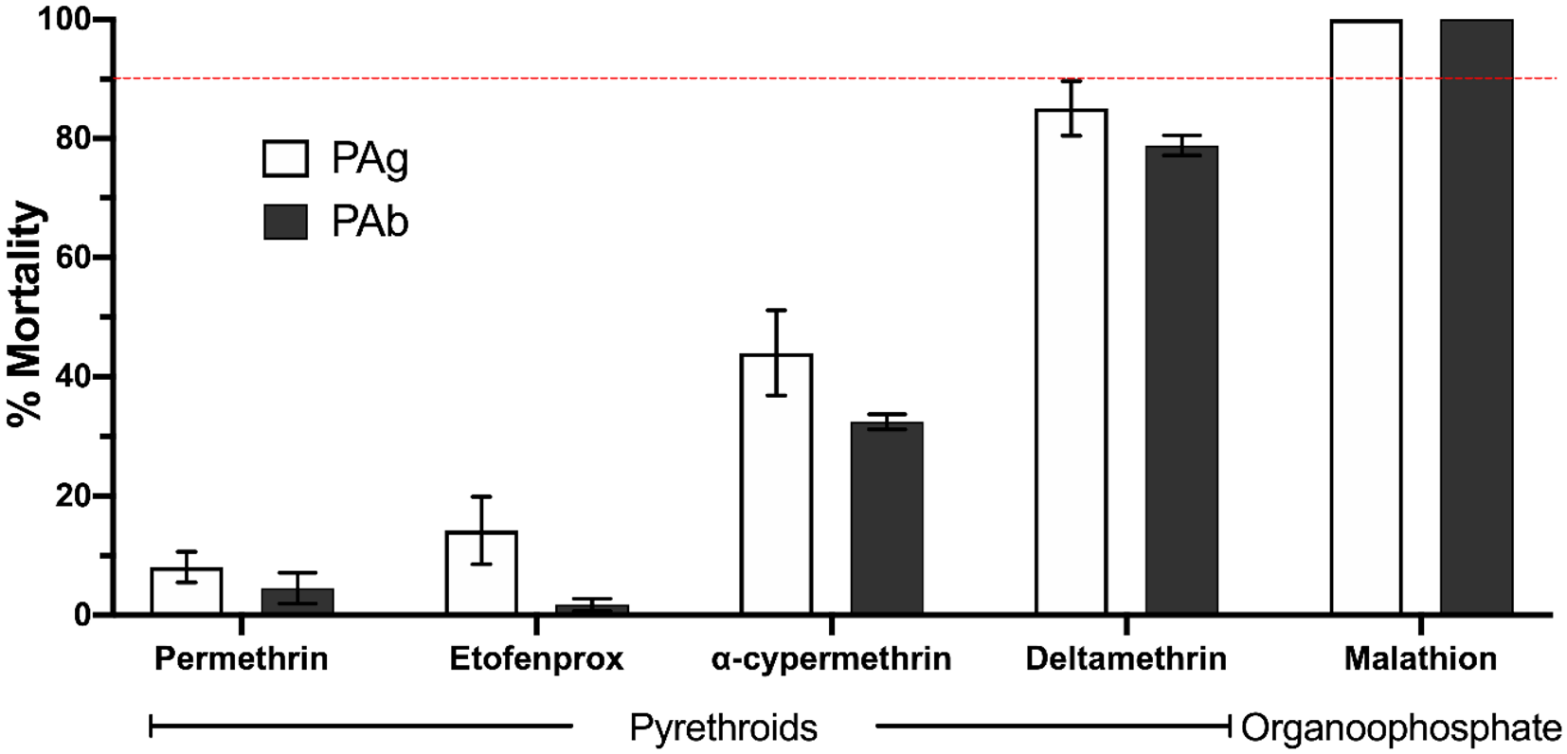
Adulticide bioassays with *Aedes aegypti* and *Aedes albopictus* from Lahore, Pakistan. PAg and PAb designate *Ae. aegypti* and *Ae. albopictus* populations, respectively. Dose-diagnostic tests with the pyrethroids permethrin (0.25%), etofenprox (0.5%), alpha-cypermethrin (0.03%) and deltamethrin (0.03%) in WHO test tubes, and the organophosphate malathion (20 µg) in bottle assays. Bars and errors indicate the mean mortality ± SEM. Populations below 90% of mortality (indicated by the red dotted line) are resistant. *Aedes aegypti* Rockefeller reached 100% mortality in all assays run in parallel.

Both species tested were resistant to the WHO diagnostic doses to the four pyrethroids, with the magnitude of resistance varying between species for different insecticides. As a whole, mortality rates were lower for permethrin and etofenprox compared to deltamethrin and alpha-cypermethrin (Fig. 2). Among pyrethroids, deltamethrin 0.03% caused the maximum mortality rates in both PAg (85.04%) and PAb (78.85%), while we observed minimum mortality rates with permethrin 0.25% for PAg (8.04%) and PAb (4.52%). In all experiments, control tubes showed no mortality, while Rock was 100% susceptible to all insecticides.

The pyrethroid with the longest estimated time for 50% of the exposed mosquitoes (*Kd*T_50_) to be knocked down was permethrin in PAg (145 min) and PAb (289 min), and the shortest *Kd*T_50_ was induced by deltamethrin in PAg (40.4 min) and PAb (46.8 min). Resistance ratios based on *knockdown* timings (*Kd*RR_50_) for the pyrethroids were higher for permethrin in PAb (8.4) and lower for deltamethrin in PAg (1.8) (Table 2).

**Table 2:** Susceptibility profile to pyrethroid adulticides in *Aedes aegypti* and *Aedes albopictus* from Lahore, Pakistan.

Pyrethroids	Knockdown time	PAg	PAb	Rock
Permethrin	K*d*T_50_ (CI95%)	145.0 (119.0–175.4)	289.9 (189.7–443.2)	34.4 (28.5–41.4)
	K*d*TRR_50_^a^	4.2	8.4	–
	K*d*T_90_ (CI95%)	336.9 (235.0–483.0)	1075.7 (506.2–2283.3)	45.2 (37.5–54.6)
	K*d*TRR_90_^a^	7.5	23.8	–
Etofenprox	K*d*T_50_ (CI95%)	120 (93.1–155.4)	246.6 (150.2–405.0)	36.8 (31.9–42.4)
	K*d*TRR_50_^a^	3.3	6.7	–
	K*d*T_90_ (CI95%)	264.5 (160.8–434.9)	607.4 (262.8–1403.9)	48.7 (40.7–58.3)
	K*d*TRR_90_^a^	5.4	2.3	–
Alfa-cypermethrin	K*d*T_50_ (CI95%)	56.1 (46.3–67.8)	61.0 (49.6–74.8)	21 (15.7–28.5)
	K*d*TRR_50_^a^	2.7	2.9	–
	K*d*T_90_ (CI95%)	101.6 (79.7–129.6)	107.5 (83.0–139.3)	30 (23.5–38.4)
	K*d*TRR_90_^a^	3.4	3.6	–
Deltamethrin	K*d*T_50_ (CI95%)	40.4 (36.6–45.6)	46.8 (38.6–56.7)	22.3 (17.8–27.9)
	K*d*TRR_50_^a^	1.8	2.1	–
	K*d*T_90_ (CI95%)	63.9 (56.6–72.2)	71.2 (59.0–86.1)	29.1 (22.9–37.0)
	K*d*TRR_90_^a^	2.2	2.4	–

^a^Resistance ratios (RR) were calculated based on *Ae. aegypti* Rockefeller strain (Rock).

### Mechanisms of resistance

#### Synergistic assay

We tested if the synergist PBO would increase mortality to the pyrethroid permethrin to evaluate the possibility of metabolic resistance mechanisms involvement in permethrin resistance. Permethrin (0.25%) caused 22.3% mortality in PAg and 19.8% in PAb, in this assay, while when pre-exposed to 4% PBO, the mortality increased to 100% and 50% in PAg and PAb, respectively. There was no mortality in the control and PBO-only conditions.

#### Expression analyses

**Figure 3:**
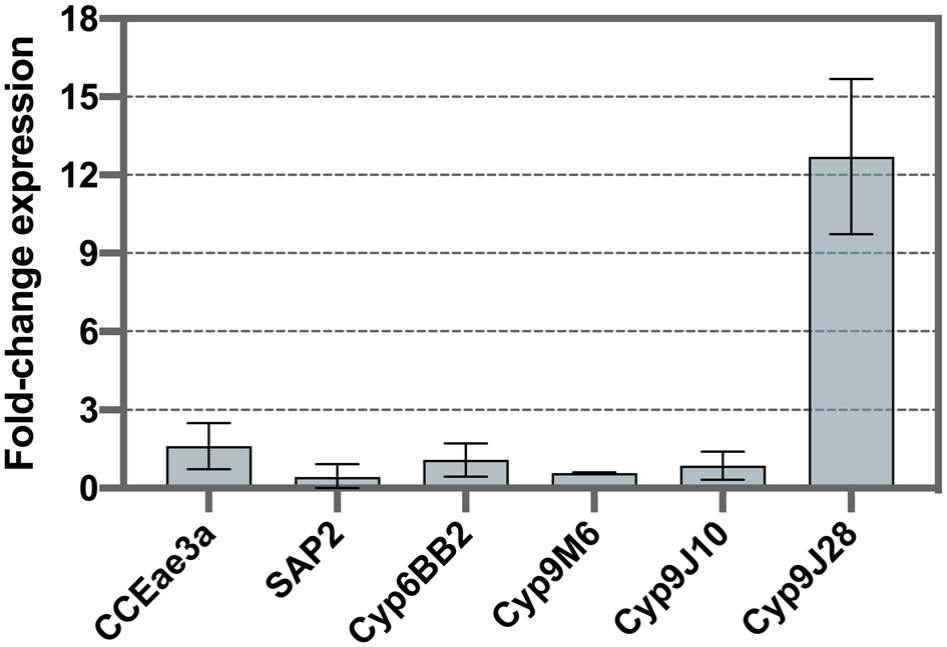
Fold-change expression of genes related to metabolic resistance of insecticides in *Aedes aegypti* from Lahore. The gene *Rps14* was used as a normalizing reference gene, and fold-change was relative to the Rockefeller strain. Means with standard deviations are represented.

We assessed the levels of expression of five genes related to the metabolism of neurotoxic insecticides (*CYP9M6, CYP9J10, CYP9J28, CYP6BB2,* and *CCEAe3a*) and a gene of sensory appendage protein (*SAP2*) in the whole-body of PAg adult females, in comparison to the housekeeping gene *Rps14*. Comparing with the profile exhibited by Rock, the only gene with a significant relative fold change expression was the MFO P450 gene *CYP9J28*, 12.7 × more expressed in PAg (Supplementary Table S1). The *SAP2* was nearly fourfold underexpressed in PAg than Rock. The fold change expression of these genes is found in Fig. 3, and more detailed data in Supplementary Table S1.

#### Kdr genotyping and sequencing

##### *Aedes aegypti*

 We genotyped 45 PAg individuals for four *kdr* mutations: V410L (IS6 segment), S989P and V1016G (IIS6 segment), and F1534C (IIIS6 segment) with TaqMan SNP assay. All samples were similar to a wild type for the three SNPs in the IS6 and IIS6 Na_V_ segments (V410, S989 and V1016). HRM analyses for the IIS6 segments were run for the exons 20 and 21 in independent reactions and indicated monomorphic sequences in exon 20 (N = 40 samples) and two variants in the exon 21 (N = 44 samples), though the distinct variant contained only one sample (#PAg_22). Sequencing of the exons 20 to 21 with the intron in between revealed two haplotypes: PAg-2s6_1 and PAg-2s6_2 (GenBank accession numbers MT707209 and MT707210, respectively). The polymorphisms were found only in the intron and justified the distinct variant observed in the HRM analysis for the #Pag_22 sample (Supplementary Figure S2). Sequences spanning exons 20–21 of the *Na*_*V*_ gene in *Ae. aegypti* are phylogenetically divided into two groups, clades A or B^23^. In this context, both PAg haplotypes belonged to the clade B, as revealed when aligned with homologous sequences available on the GenBank (NCBI). PAg-2s6_1 was similar to a haplotype observed in *Ae. aegypti* populations from the Americas, Africa, Asia, and Australia (MN602762). PAg-2s6_2 was similar to haplotypes obtained in samples from Brazil, Australia and Kenya (MN602775) and Vietnam (MG257775, LC036556). This haplotype is also similar to the sequence of the reference lab strain LVP (MK977832) originally collected in Sierra Leone in the 1930s (Supplementary Figure S2).

**Figure 4:**
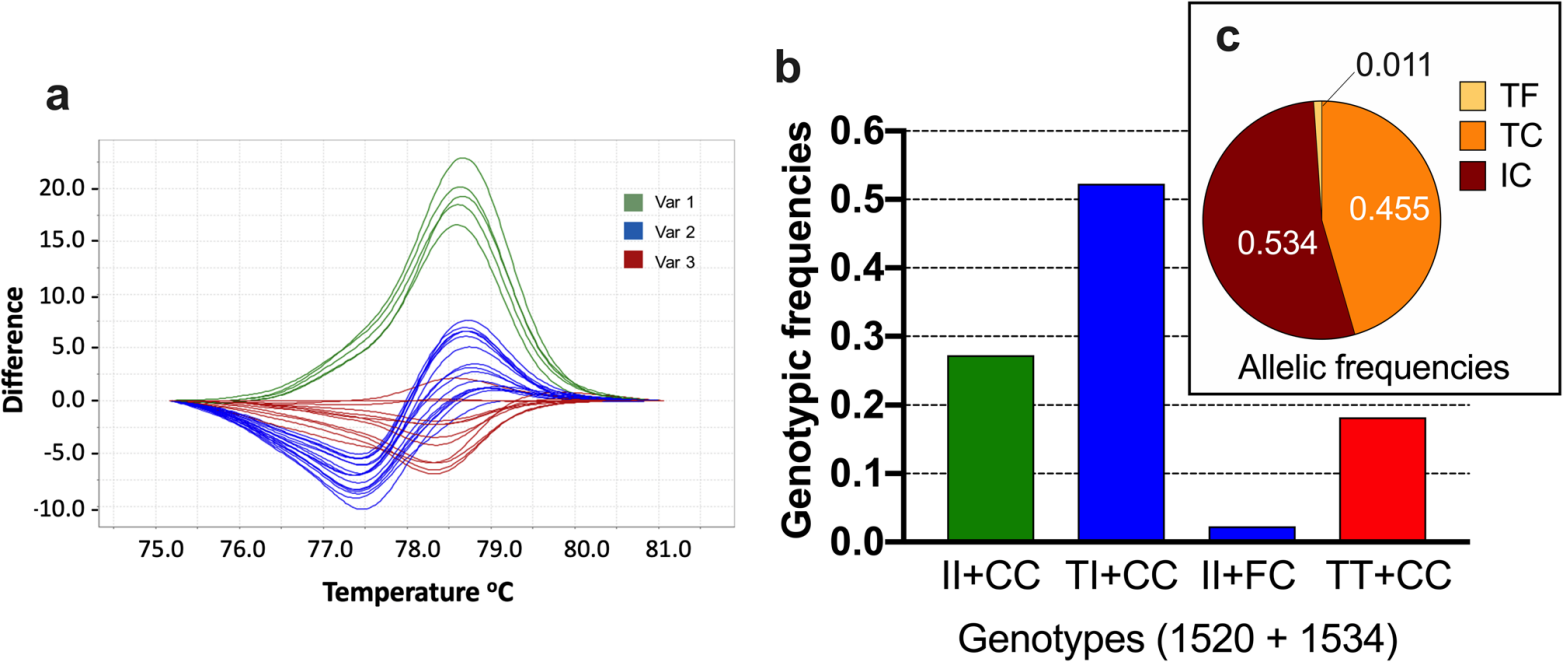
*Kdr* genotyping in the Na_V_ IIIS6 segment in *Aedes aegypti* from Lahore, Pakistan. (**a**) The high-resolution melting analyses (HRM) difference plot, where each line represents a sample, is grouped into three variants. Sequencing of some samples of each variant, summed with TaqMan genotyping of the F1534C SNP indicated their genotypes, which then rendered the genotypic (**b**) and allelic frequencies (**c**), considering the 1520 and 1534 sites.

For the IIIS6 segment, however, 44 samples were *kdr* homozygous (1534 C/C), and one sample (that same #PAg_22) was heterozygote (1534 F/C), as obtained by TaqMan SNP assay for the F1534C SNP. In addition to this TaqMan assay, an HRM analysis in part of the exon 31 for this segment determined three variants (Fig. 4a), which when sequenced (see below), indicated variation in the 1520 (T1520I) in addition to the 1534 (F1534C) site. The genotypes were then determined as 1520 T/T + 1534 C/C (*kdr* homozygous at 1534) in 18.2% samples, 1520 I/I + 1534 C/C (*kdr* homozygous in both sites) in 27.3% samples, and 1520 T/I + 1534 C/C or 1520 T/T + 1534 F/C (heterozygous at 1520 or 1534 sites) in 54.5% samples (Fig. 4b). The exon 31 sequencing of the aforementioned #PAg_22 indicated its genotype as 1520 T/T + 1534 F/C. Summing up all of this information, the allelic frequencies in PAg were: 53.4% (IC), 45.5% (TC) and 1.1% (TF) (Fig. 4c). Three haplotypes were identified in the IIIS6 segment out of 13 sequenced samples (Supplementary Figure S2): the wild-type (PAg_3s6-1) without any nonsynonymous substitution, and two *kdr* haplotypes: one with the 1534C (PAg_3s6-2) and the other with both 1520I and 1534C *kdr* mutations (PAg_3s6-3). The PAg_3s6-1 haplotype was observed only under heterozygosis (overlapped peaks in the electropherogram) with PAg_3s6-2, confirming the #PAg_22 genotype (1520 T/T + 1534 F/C). We aligned these haplotypes with 69 other homologous sequences available on the NCBI GenBank and found that PAg_3s6-1 was similar to the wild-type haplotype described in *Ae. aegypti* populations from all continents. Likewise, PAg_3s6-2 was similar to the 1534C *kdr* sequences observed worldwide. PAg_3s6-3, with the double mutation 1520I + 1534C, was similar to sequences from Thai and Indian populations (Supplementary Figure S2).

##### *Aedes albopictus*

 HRM analyses for each of the exons 20 and 21 showed two variants in PAb (N = 45 samples) in both reactions. When sequenced, four SNPs were found in the exon 20, indicating five haplotypes (Supplementary Figure S2). These observed SNPs were three nonsynonymous substitutions and the mutation L882M. A Blast search with the exon 20 of these five haplotypes matched with identical sequences of  *Ae. albopictus* populations from Brazil and China (Supplementary Figure S2). The L882M substitution was also present in a sample from India (MF776970)^32^, although in a haplotype distinct to the Pakistani herein observed.

Concerning the exon 31, we obtained a 257 bp fragment from 13 samples, in which a total of six SNPs were identified, all synonymous substitutions. The sequences of eight haplotypes are available in (Supplementary Figure S2) (GenBank accession numbers: MT740758-MT740765). The haplotypes Pab_3s6-1 and Pab_3s6-2 were identical to sequences from *Ae. albopictus* from China and Malaysia, available on NCBI GenBank (Supplementary Figure S2).

## Discussion

There are few insecticide resistance studies in Pakistani *Aedes spp*^12,13,15,16,33–40^, in a scenario where vector control on its own has been one of the neglected aspects of arthropod-borne infections. There were interesting projects about IR status and mechanisms selected in *Anopheles* mosquitoes during the late 1970s and early 1980s^39,41–43^. Nevertheless, the studies on this particular area were discontinued due to its geopolitical situation and other factors. Lahore, the second-most populous city in Pakistan, has faced various dengue outbreaks in the last decade. Before dengue vector control, several chemicals from pyrethroid, organophosphates, and organochlorine classes were employed in malaria vector control programs. They included deltamethrin, permethrin, malathion, DDT, among others. Here we evaluated the insecticide resistance status of *Ae. aegypti* and *Ae. albopictus* from Lahore to the larvicides temephos (organophosphate) and pyriproxyfen (IGR), as well to the adulticides permethrin, etofenprox, alpha-cypermethrin and deltamethrin (pyrethroids), and malathion (organophosphate). We evidenced that these species were still susceptible to both organophosphates and that the IGR is a suitable additional compound to be used in the region. On the other hand, *Ae. aegypti* and *Ae. albopictus* were resistant to all types of pyrethroids here evaluated. In addition to *kdr* mutations, an overexpressed P450 *cyp* gene might be playing a role in resistance to pyrethroids.

Temephos has been employed in Punjab, Pakistan, against *Aedes* spp. since 2011–2012, and resistance to this larvicide was detected in *Ae. aegypti* from several cities of that district, including Lahore, collected in 2016^15^. Surprisingly, the LC_50_ of temephos in *Ae. aegypti* from this same city was around 13X lower in our study than that samples collected 2 years earlier (0.08^15^ against 0.006 µg/mL). A decrease in temephos resistance was indeed observed in laboratory lines maintained in the absence of selection pressure as well as in natural populations, some years later without the employment of temephos^44^ (*Rahman *et al. ^45^), likely due to a substantial fitness cost associated with physiological changes selected for resistance^46^. However, this decrease can be relatively slight and requires several generations or years in the field without selection pressure, and to our knowledge, temephos is still applied in Lahore. Further studies with new collections are necessary in order to understand this phenomenon better. In the case of *Ae. albopictus* from Lahore, there were records of resistance to the organophosphates larvicides chlorpyrifos, profenofos, and triazophos^47^, while in our results *Ae. albopictus* presented a temephos LC_50_ value similar to that of *Ae. aegypti*. Concerning the adulticide malathion, both *Ae. aegypti* and *Ae. albopictus* populations from Lahore were susceptible in collections performed in 2015^36^, and maintained this status as we observed here.

The IGRs emerged as a prominent alternative to neurotoxic larvicides^48^. The chitin synthesis inhibitor compounds like diflubenzuron and buprofezin have been tested in *Ae. aegypti* from Lahore, with diflubenzuron causing higher mortality at the pupal stage while buprofezin resulting in more larval deaths^16^. Both *Ae. aegypti* and *Ae. albopictus* we evaluated here showed 100% of adult emergence inhibition against the analog of juvenile hormone IGR, pyriproxyfen, with maximum mortality at the pupal stage, as previously observed in 2015^40^. Also, as an alternative to chemicals, the biolarvicide *Bti* caused 100% mortality in the *Aedes* field populations from the same city^16,34^. Although resistance to pyriproxyfen and other IGRs is not common, there are reports of Brazilian and Malaysian populations resistant to pyriproxyfen and methoprene, respectively^49,50^. Therefore, although IGRs have emerged as a promising alternative to neurotoxicants, their effectiveness must be continuously monitored.

Although pyrethroids are not used as larvicides, we performed a rapid test with permethrin and deltamethrin, adopted as a preliminary method to indicate pyrethroid resistance in mosquito larvae^51^. Based on a similar assay, we did not evidence resistance to pyrethroid in Pakistani *Ae. aegypti* and *Ae. albopictus* larvae. However, both species were resistant to all tested pyrethroid adulticides. Levels of mortality were under 20% in adult bioassays with the pyrethroid type I permethrin, though around 80% to the type II pyrethroid deltamethrin. Resistance to permethrin and deltamethrin was previously recorded in *Ae. aegypti* from Lahore, collected in 2010^35^. In 2015, both *Ae. aegypti* and *Ae. albopictus* from four towns in the Lahore district were found to be resistant to permethrin, lambda-cyhalothrin, and deltamethrin, in addition to DDT^36^.

Deltamethrin and permethrin resistant *Ae. aegypti* populations from Lahore showed a significant increase in mortality after being treated with synergist PBO^15^. Our study corroborated those findings by observing that a pre-exposure to 4% PBO increased mortality to permethrin (from 22 to 100% in *Ae. aegypti* and from 19.8% to 50% in *Ae. albopictus*), suggesting an influence of P450 detoxifying enzymes. Indicative of metabolic resistance in PY resistant *Ae. aegypti* from Lahore was previously shown by biochemical analysis, which indicated higher quantities of esterases, MFOs, GSTs and AchE^33^. Here we evaluated the expression of genes previously related to metabolic resistance, such as the P450 MFOs (*CYP9J28, CYP9M6* and *CYP6BB2*) and the carboxylesterase *CCEAe3a*^52–54^. This trend has been identified in resistant *Aedes* population from Southeast Asia^29,54–56^, the Caribbean^57^, Central^58^, and South America^59^. Of them, we found *CYP9J28* 12-fold overexpressed in *Ae. aegypti* from Lahore. Its role has already been shown in vivo, when the transgenic expression of *AaegCYP9J28* increased pyrethroid-resistance in *D. melanogaster*^60^. As far as the overexpression of carboxylesterase genes in *Ae. aegypti* is concerned, they are more associated with OP resistance^61^. Indeed, as we did not observe resistance to the OPs temephos and malathion, it makes sense that the *CCEAe3a* gene was not overexpressed in our samples from Lahore.

*Knockdown* resistance mutations in voltage-gated sodium channel (Na_V_) alone or in combinations have been reported from several *Ae. aegypti* populations from Southeast and South Asian countries including Malaysia^62^, Indonesia^63^, Thailand^64^, Singapore^65^, Myanmar^66^, India^67^, Sri Lanka^68^, Laos^30^, China^69,70^ and Saudi Arabia^71^. Here the *Ae. aegypti* Pakistani populations were genotyped to several *kdr* SNPs: V410L, described in populations from Latin America^72^, P989S and V1016G from Asia^54,66,73^ and F1534C, present in all continents^23,74,75^. Mutations on these sites alone or in combination are expected in populations resistant to pyrethroids and DDT, as recently reviewed^21^. We did not detect these classical SNPs at IS6 (V1014L) and IIS6 (S989P and V1016G) Na_V_ segments, nor any additional nonsynonymous substitutions in the IIS6 segment. Interestingly, the sequences of this segment in worldwide *Ae. aegypti* populations are divided into two clades, A and B, distinguished mostly by indels in the intron between exons 20 and 21. Besides, all IIS6 *kdr* mutations described so far are in haplotypes from clade A^23^. Here all IIS6 sequences of *Ae. aegypti* belonged to clade B. On the other hand, the 1534C *kdr* mutation in the IIIS6 segment was present in all *Ae. aegypti* samples from Lahore here evaluated, as indicated by TaqMan genotyping assay specific for the F1534C SNP and confirmed by sequencing. Moreover, HRM analyses displayed a distinct profile that verified the presence of the SNP T1520I. Summing up TaqMan, HRM, and sequencing analyses, we evidenced two *kdr* haplotypes: T1520 + 1534C (45.5%) and the double *kdr* 1520I + 1534C (53.4%). The haplotype without *kdr* T1520 + F1534 (1.1%) was present in only one sample. This partially explains the resistance observed in the Pakistani *Ae. aegypti* population to the pyrethroids. The F1534C *kdr* has been described in *Ae. aegypti* populations from several Asian countries^76^. In PY-resistant Indian *Ae. aegypti* populations, a PCR–RFLP analysis followed by sequencing showed the presence of three haplotypes [T1520 + F1534 (21%), T1520 + 1534C (66%), and 1520I + 1534C (13%)] as we have reported in our findings. It was also observed that the F1534C mutation always occurred independently while T1520I was always found in association with F1534C^14,23^.

In the case of *Ae. albopictus*, HRM analyses identified distinct variants in the analyses of both IIS6 and IIIS6 Na_V_ segments. However, they all accounted for synonymous substitutions, as revealed by sequencing, except for one nonsynonymous in the IIS6 segment: L882M. Likewise, several mutations in PY-resistant *Ae. albopictus* populations from India were also reported^32^. Chinese *Ae. albopictus* presented a different mutation (F1534S) in pyrethroids resistant populations^70^. It is noteworthy that the diversity of haplotypes in *Ae. albopictus* was higher than *Ae. aegypti*, as expected since it is native to Asia while *Ae. aegypti* is an invasive species^77^. Consequently, lower diversity is expected in this species, compared to the autochthonous Asian tiger mosquito.

## Conclusion

*Ae. aegypti* and *Ae. albopictus* from Lahore, Pakistan, were resistant to pyrethroids while susceptible to organophosphates and the IGR. Among compounds we tested herein, both larvicides temephos and pyriproxyfen, as well as the adulticide malathion, should be the most effective against both species. Synergist PBO increased mortality against permethrin, indicating the participation of metabolic resistance mechanisms. In fact, the P450 gene *CYP9J28* was overexpressed in *Ae. aegypti*. Also, the *kdr* mutations T1520I and F1534C were present under high frequencies. Therefore, resistance to pyrethroids in *Ae. aegypti* from Lahore is likely related to multiple physiological mechanisms. These results' implications may be discussed with authorities responsible for Lahore's vector control actions, aiming to improve strategies against *Aedes* in Pakistan.

## Methods

### Sample collection and laboratory rearing

**Figure 5:**
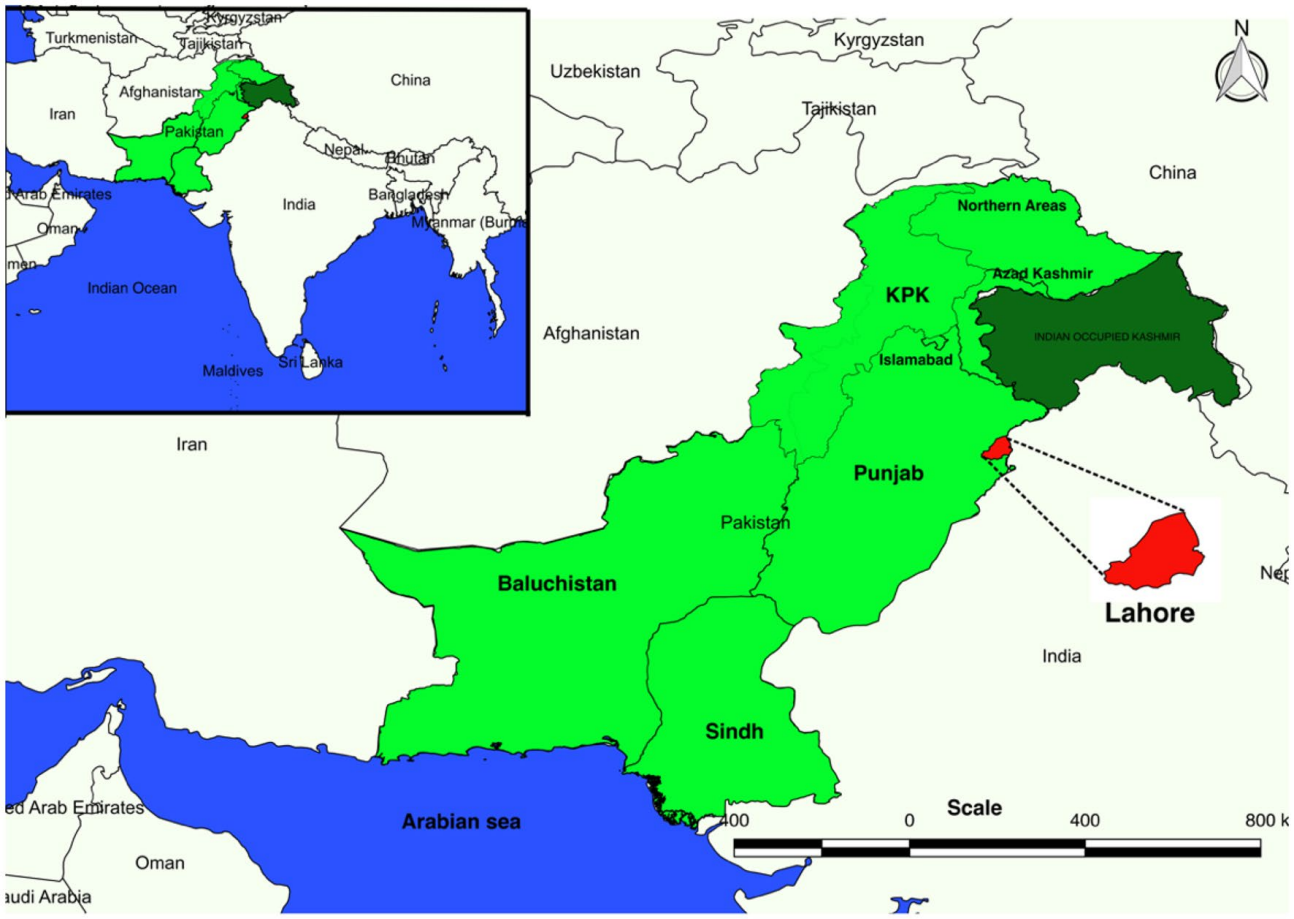
Political map of Pakistan, divided into four provinces, with Lahore magnified in red. The map was generated with a free and open- source software QGIS version 2.18.24 (GNU General Public License), developed by the Open Source Geospatial Foundation Project (http://qgis.org).

We collected larvae of *Aedes aegypti* and *Aedes albopictus* from different Union Councils (UCs)/neighborhoods of Mughulpura and airport areas of Lahore district with the help of sanitary agents and health workers recruited by the local health department for this purpose (Fig. 5).

We visited different domestic and industrial areas. Larvae mainly infested uncovered or partially covered water storage tanks, buckets, and small pots made from various materials, including plastic, steel, copper, and cement, which contained water and were located in shaded places. Larvae were brought to the insectary of the Department of Zoology, GC University Lahore, identified into species level with the help of morphological characters^78^, and provided with fish food (Tetra- Marine Granules, Tetra). Emerged pupae were shifted to clean and disinfected cages, and adults were provided with a 10% sugar solution. Adults were starved overnight but allowed to feed on blood using an artificial feeder, Hemotek (PS-6 System, Discovery Workshops, Accrington, UK). Eggs of the F1 generation were shipped to Laficave, IOC/FIOCRUZ, Brazil, following the formalities of the Brazilian Ministry of Agriculture and the relevant authorities in Pakistan. Colonies were raised to F2 and F3 generations in our insectary at Fiocruz in the same fashion as mentioned before, while F1 males were preserved at − 20 °C for genotyping. The rabbit blood utilized to feed the mosquitoes was kindly provided by the Institute of Science and Technology in Biological models (ICTB), Fiocruz, under the license CEUA L-004/2018, approved by the Ethics Committee for the Use of Animals in Research of Oswaldo Cruz Institute (CEUA-IOC).

With the purpose of maximizing growth synchronization, we put the *Aedes* eggs to hatch in 1 L dechlorinated water, and after 3–4 h, 500 larvae were transferred to separate trays and grown to L3/L4 stage under standard lab conditions of temperature 26 °C ± 1 °C, light: dark 12 h: 12 h period and 70 ± 5% relative humidity^46^. The *Ae. aegypti* Rockefeller strain, the international reference for susceptibility to insecticides and vigor under laboratory conditions^79^, was reared in parallel and tested in all experiments as an internal control. We have referred to *Ae. aegypti* Rockefeller as Rock, and *Ae. aegypti* and *Ae. albopictus* Lahore populations as PAg and PAb, respectively.

### Bioassays with larvae

#### Temephos

We followed the dose–response WHO protocol to calculate the resistance ratios (RR) to temephos in the evaluated populations^80^. Temephos technical grade (Pestanal Sigma-Aldrich) was dissolved in ethanol to give a series of concentrations ranging from 0.002 to 0.018 mg/L in order to obtain mortality from 5 to 99%, as recommended for probit analysis. Each concentration and a negative control condition (containing 300 µL of ethanol only) was replicated four times, each containing 100 mL solution with ~ 20 late L3 or early L4 larvae, in a disposable plastic cup of 150 mL capacity. Mortality was calculated 24 h after initial exposure. The tests were performed three times with both PAg and PAb populations simultaneously, always in parallel to Rock as an internal control.

#### Pyriproxyfen

For the IGR pyriproxyfen, we performed dose-diagnostic assays. Each assay contained eight treatment replicas with pyriproxyfen (Sigma-Aldrich) at a diagnostic concentration of 0.3 μg/L. This dosage was previously obtained, as twice the CL_99_ for Rock^49^. Besides, we included six control replicas (with 1 mL of the solvent instead of the IGR solution) in a 250 mL solution. A 300 mL disposable, transparent plastic cup was used for each replica, into which we added 10 L4 larvae. To nourish the larvae, we introduced 15 mg of fish food (Tetramarine Granules, Tetra) at the start of the assay and 10 mg on the fourth day of the experiment. All the cups were covered with gauze to avoid eventual adult escaping. Mortality and life stage transformation were cumulatively recorded bi-weekly. Parallel assays were done with Rock. Data was recorded until all individuals in the control condition emerged into adults. Assays were performed three independent times, under standard environmental conditions of temperature (26 °C ± 1 °C), light: dark regiments (12 h: 12 h), and relative humidity (75 ± 5%). Unlike neurotoxic insecticides, for IGRs, mortality itself is not the critical parameter to evaluate, but the index of Adult Emergence Inhibition (AEI). Populations are considered to be resistant when AEI is lower than 90%. In addition to AEI, we recorded the mortality observed at each developmental stage: larvae, pupae, and adult (adults that remained attached to the exuviae and died).

#### Susceptibility Index of Aedes larvae against pyrethroids

We adopted a simplified knockdown assay^51^ to evaluate susceptibility to the *knockdown* effect to type I (permethrin) and type II (deltamethrin) pyrethroids in larvae. We prepared solutions with technical grades permethrin (Pestanal, Sigma-Aldrich) and deltamethrin (Pestanal, Sigma-Aldrich) by dissolving each in 1 mL acetone and then ethanol to make 250 ppm stock solutions. From this, we prepared a 20 mL solution for each pyrethroid, with 10 replicas for two concentrations (0.1 ppm and 0.4 ppm) in H_2_O. Two negative control cups with ethanol (32 µL) in 20 mL of H_2_O were run in parallel. We used one L4 larva per replica and registered the knockdown every 5 min, for half an hour. The *Kd*T_50_, i.e., the time when 50% of the larvae were knocked down, was scored according to these categories: 1 (0–5 min), 2 (6–10 min), 3 (11–15 min), 4 (16–20 min), 5 (21–30 min) or 6 (> 30 min). The susceptibility index (SI) for each species against deltamethrin and permethrin was obtained by multiplicating the *Kd*T_50_ categories of both concentrations (0.1 and 0.4 ppm). Population with the lower SI is considered more sensitive. The obtained SI values were the mean of three independent assays, with Rock in parallel as an internal control in all of them.

### Adulticides

#### Pyrethroids

We followed the WHO-like tube tests procedure, with modifications^81,82^ for the tarsal contact tests with insecticides-impregnated filter papers. Papers (Whatman grade 1) were impregnated with diagnostic concentrations of four pyrethroids following WHO standard protocol and dosages: deltamethrin 0.03%, permethrin 0.25%, etofenprox 0.5% and alpha-cypermethrin 0.03%^81^. Solutions were prepared from technical grade insecticides (Pestanal Sigma-Aldrich) in acetone and silicone oil (used as a carrier) and evenly applied to a filter paper (Whatman grade 1). Papers were allowed to air dry for 72 h before use. Papers impregnated with just solvent were used as the control. In each assay, we put 15–20 female mosquitoes, 3–5 days old, non-blood-fed, in a resting tube (tube without insecticide) to acclimatize for 30 min. After that, they were gently blown into tubes having insecticides-impregnated papers, and knockdown was checked every 5 min for 2 h. This differed from the WHO protocol^81^, which recommends exposure time of 60 min. In the case of Rock, mortality was checked every two minutes. After they were exposed to insecticides, mosquitoes were shifted back to resting tubes, provided 10% sugar water, and mortality evaluated after 24 h.

#### Organophosphate

For the OP malathion bioassays, we used CDC bottle tests^83^, impregnating 250 mL glass bottles (Wheaton) with 20 µg/mL malathion (Cheminova Brasil Ltda, São Paulo) dissolved in acetone. For impregnating the bottles, 1 mL of the insecticide solution was distributed evenly to all parts of the bottle, including its cap, and left to air dry for at least 24 h before the test. In each bottle, 20–25 female mosquitoes, three to five days old, non-blood fed, were left for one hour, and mortality was recorded. Bottles impregnated with 1 mL acetone were used as control. This experiment was done in four replicates, three separate times. Standard conditions of temperature, humidity, and light–dark periods were maintained throughout the assays, as described earlier.

#### Synergist assay

In order to evaluate the occurrence of metabolic resistance mechanisms to the pyrethroid type I permethrin, we carried out WHO bioassays with the synergist PBO (Piperonyl Butoxide)^84^. This substance inhibits the action of Multi-Function Oxidases (MFOs) and can revert resistance^85^. For this purpose, papers were impregnated with technical grade PBO (Endura) and permethrin (Pestanal, Sigma-Aldrich), as described earlier. The test was composed of four conditions: (i) exposure to 4% PBO, (ii) a pre-exposure to 4% PBO and then to 0.25% permethrin, (iii) exposure to 0.25% permethrin only, and (iv) exposure only to solvent control. The susceptible strain, Rock, was carried out parallelly. Each assay consisted of four tubes, and each tube contained 20–25 female mosquitoes, 4–6 days old, non-blood-fed. All exposures lasted 1 h, after which mortality was checked in each assay. Mosquitoes were then transferred to resting tubes and provided 10% sugar solution. The number of dead mosquitoes was counted after 24 h, according to the standard criteria of WHO^81^.

#### Calculations

In the case of both pyrethroids and organophosphates bioassays, we expected 100% mortality of the reference strain and no (zero) mortality in the control. If mortality in the control exceeded 20%, the test should be repeated. If less, Abbott's correction formula would be applied^86^. Temephos Lethal Concentrations (LC) were obtained by log x probit transformations followed by linear regression analyses^87^ with the sum of the values from the three assays. We calculated the resistance ratios (RR) by dividing the LC of both PAg and PAb by the LC of Rock, in the absence of a reference lab strain for *Ae. albopictus* at that time.

To estimate knockdown time to pyrethroids (*Kd*T_50_), readings were likewise submitted to probit analysis. Accordingly, *knockdown*-time Resistant Ratios (*Kd*T-RR_50_) were calculated by dividing *Kd*T_50_ of PAg and PAb by the *Kd*T_50_ of Rock.

### Exploration of knockdown resistance (kdr) mutations

We evaluated the nucleotide diversity in the genomic region corresponding to IS6, IIS6, and IIIS6 Na_V_ segments to investigate *kdr* mutations classically found in *Ae. aegypti* pyrethroid-resistant populations. Genomic DNA was isolated from male mosquitoes (n = 45) of both PAg and PAb with a column-based DNA extraction kit (*NuleoSpin*, Macherey–Nagel Laboratories), according to manufacturer's instructions. We did not use females to avoid eventual amplification of DNA inside their spermathecae, which would mislead interpretations about individual genotypes. Purified DNA was quantified with NanoDrop One (ThermoFisher Scientific) and diluted to 20 ng/µL in ultra-pure water.

#### SNPs genotyping

A TaqMan SNP genotyping qPCR approach was employed for *kdr* genotyping, essentially as described previously^88^, for the variations Val410Leu, Ser989Pro, Val1016Gly, and Phe1534Cys in PAg. We performed reactions independently for each SNP, consisting of 1X TaqMan Genotyping Master Mix (ThermoFisher), 1X of the respective Custom TaqMan SNP Genotyping Assay (Table 3), 20 ng of DNA and ultra-pure water *q.s*. 10 µL, run in a QuantStudio 6 Flex (Applied Biosystems), under standard conditions. The genotype callings were obtained by the online software Genotype Analysis Module V3.9 (Applied Biosystems, Thermo Fischer cloud platform). As a positive control, we used the DNA of Rockefeller strain (wild-type homozygote genotypes) in all SNP reactions, and the Rock-*kdr* strains^89^, which has the *kdr* homozygote genotypes: 410 Leu/Leu, 1016 Ile/Ile and 1534 Cys/Cys. Also, we employed a synthesized DNA fragment (gBlock, IDT) with the sequence of an Asian *kdr* haplotype (GenBank accession number MN602755)^23^ as the *kdr* homozygote genotypes in 989 Pro/Pro and 1016 Gly/Gly SNP reactions. An equimolecular quantity of Rock DNA was mixed with each *kdr* positive controls to obtain the respective heterozygote controls.

**Table 3:** Primers and probes for TaqMan SNP genotyping qPCR assays for *kdr* mutations in *Aedes aegypti*.

Na_V_ Site, segment	Assay ID^a^	Variation	Primers	Probes
410, IS6	AN2XA9W	GTA/TTA (Val/Leu)	for: GTGGCACATGCTCTTCTTCATT rev: GGCGACAATGGCCAAGATC	Val: VIC-TCGTTCTACCTTGTAAATT-NFQ Leu: FAM-TTCGTTCTACCTTTTAAATT-NFQ
989, IIS6	AH21C3C	TCT/CCT (Ser/Pro)	for: TGATCGTGTTCCGGGTATTATGC rev: CCATCACTACGGTGGCCAAAA	Ser: VIC-CCCACATGGATTCGAT-NFQ Pro: FAM- CCACATGGGTTCGAT-NFQ
1016, IIS6	AHX1KC9	GTT/ GGT (Val/Gly)	for: CGTGCTAACCGACAAATTGTTTCC rev: TTGGACAAAAGCAAGGCT	Val: VIC-AGAAAAGGTTAAGTACCTGTGCG-NFQ Gly: FAM- AGAAAAGGTTAAGTACCTGTGCG-NFQ
1534, IIIS6	AHWSL61	TTC/TGC (Phe/Cys)	for: TCGCGAGACCAACATCTACATG rev: GATGATGACACCGATGAACAGATTC	Phe: VIC-AACGACCCGAAGATGA-NFQ Cys: FAM-ACGACCCGACGATGA-NFQ

^a^ Identification of the customized TaqMan SNP Genotyping Assay (Thermo Fischer).

#### HRM reactions

**Figure 6:**
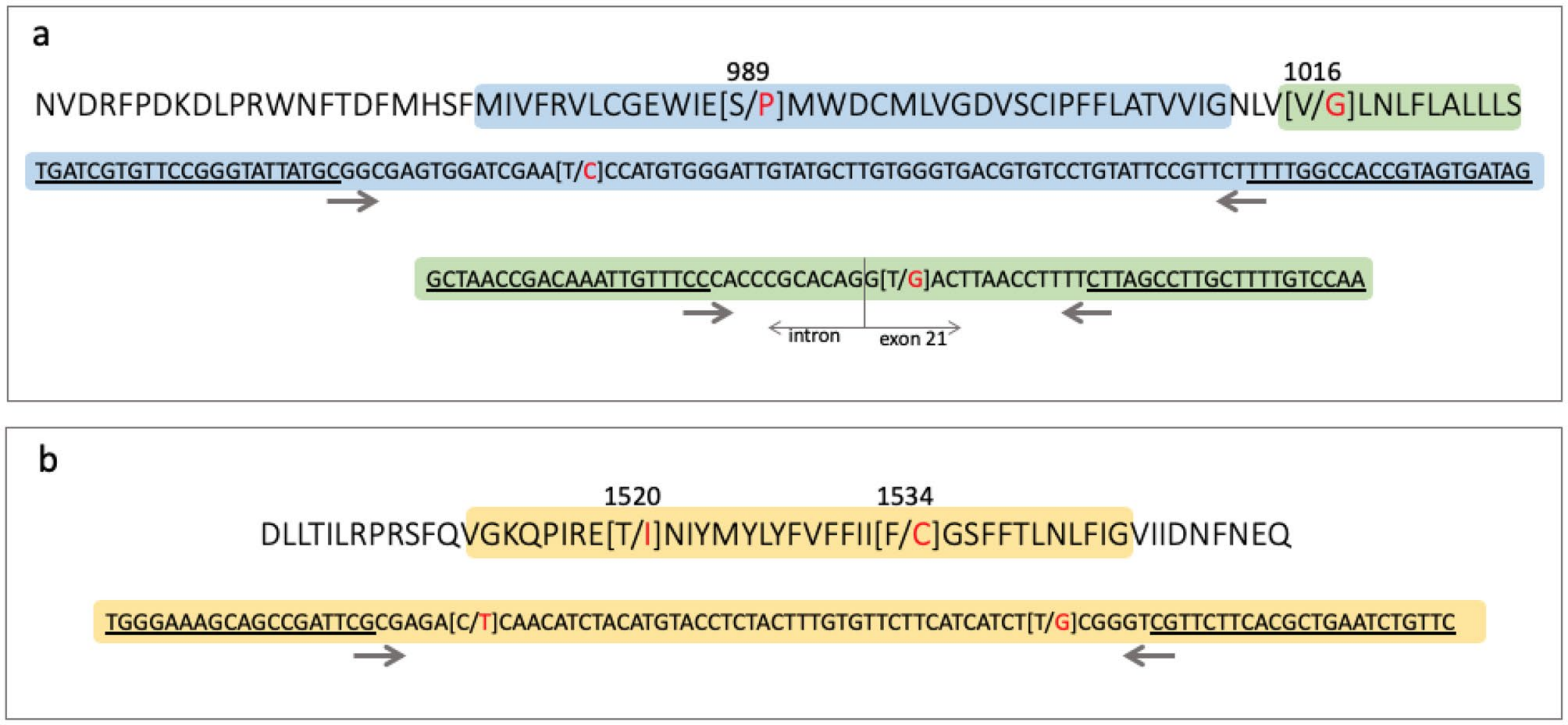
Representation of the IIS6 (**a**) and IIIS6 (**b**) Na_V_ segments with their respective amino acid (above) and nucleotide (bellow) sequences. The SNPs at 989, 1016, 1520, and 1534 sites are indicated inside brackets with the mutant alternative in red. The shaded colors indicate the amplified sequences in the HRM reactions, with their respective primer sequences underlined and orientation following the arrows.

To search for possible SNPs beyond the classical mutations tested with TaqMan qPCR, we developed a high-resolution melting analysis (HRM) for the genomic regions in the IIS6 and IIIS6 Na_V_ segments of PAg and PAb. We performed three HRM reactions, each for part of the *Na*_*V*_ gene exons 20, 21 (IIS6), and 31 (IIIS6) (Fig. 6). We focused on these exons because the known *kdr* sites 989, 1016, and 1534 are respectively placed in the exons 20, 21, and 31 of the *Ae. aegypti NaV* gene. Reactions were performed with 1X MeltDoctor HRM Master Mix kit (Thermo Fischer), 0.3 µM of each primer (Table 4), 20 ng DNA, and ultra-pure water *q.s*. 10 µL. The thermocycling program followed the standard qPCR conditions with an additional HRM step in a QuantStudio 6 (Applied Biosystems). The HRM curve analyses were performed with the QuantStudio Real-Time PCR Software v1.3 (ThermoFisher), which grouped the samples into distinct variants. To determine the variant genotypes, we sorted at least three samples of each variant to be sequenced.

**Table 4:** Primers for HRM analyses in *Aedes aegypti* and *Aedes albopictus*.

Na_V_ segment	Targeted sites	Primer sequences	Species
IIS6	Exon 20	for: TGATCGTGTTCCGGGTATTATGC	Both
		rev: CCATCACTACGGTGGCCAAAA	Both
	Exon 21	for: GCTAACCGACAAATTGTTTCC	*Ae. aegypti*
		for: GAATGCTTTCTCCCCCAAAC	*Ae. albopictus*
		rev: TGGACAAAAGCAAGGCTAAG	Both
IIIS6	Exon 31	for: TGGGAAAGCAGCCGATTCG	Both
		rev: GAACAGATTCAGCGTGAAGAACG	Both

All primer sequences are in the 5′-3′orientation.

#### DNA sequencing

The IIS6 and IIIS6 corresponding genomic regions were amplified with Phusion High-Fidelity DNA Polymerase PCR kit (New England BioLabs) in a 25 µL reaction, containing 1X Phusion HF buffer, 200 µM dNTP, 3% DMSO, 0.4 U Polymerase, 0.5 µM of each primer, 40 ng DNA and H_2_O *q.s.* 20 µL. The primers employed were *5para3*: ACAATGTGGATCGCTTCCC x Na_V__E21R: GCAATCTGGCTTGTTAACTTG for the IIS6 segment in both species and *31P*: TCGCGGGAGGTAAGTTATTG x *31Q*: GTTGATGTGCGATGGAAATG for the IIIS6 segment in *Ae. aegypti*, and *31F*: GATCGCGGGAGGTAAGTT X *31R*: CCGTCTGCTTGTAGTGATCG in *Ae. albopictus* (all primers described in 5′ to 3′orientation). Thermocycling conditions were 98 °C/30" for initial polymerase activation, followed by 35 cycles of 98 °C/30" for double-strand denaturation, 60 °C (in IIS6 reactions) or 61 °C (in IIIS6 reactions)/15" for primer annealing, and 72 °C/30" for enzyme extension. The PCR products were purified with magnetic beads Agencourt AMPure XP (Beckman Coulter), following manufacturer instructions. Purified amplicons (1 µL) were submitted to a Sanger sequencing reaction with the kit BigDye Terminator v3.1 (ThermoFisher), with 1 µM of one of the primers, according to manufacturer's protocol, and followed to the FIOCRUZ platform of DNA sequencing. Each sample was sequenced in both strands. Sequences were analyzed with Geneious v. Prime^90^ and submitted to GenBank (accession numbers: MT740753-MT740765 and MT707209-MT707210). We compared our sequences with those available in the GenBank (NCBI Blast) to check their similarity with sequences from worldwide populations.

### Expression analysis of genes related to detoxification of insecticides

We evaluated the expression profiles of genes previously associated with resistance in *Aedes* populations from South-east Asia in PAg populations: three were from the *CYP9* family (*CYP9M6*, *CYP9J10,* and *CYP9J28*) and one from *CYP6* (*CYP6BB2*). The expression profile of one carboxylesterase (*CCEAe3a*) and one sensory appendage protein (SAP2) was also investigated^31,91,92^. The expression levels were relative to the housekeeping gene of the ribosomal protein S14 (*RpS14*)^93^.

Five-day-old female mosquitos were pooled in a tube. For each population, we used four pools as biological replicates per population. In order to isolate RNA, these mosquitoes were macerated in 300 µL TRIzol (Invitrogen, CA, USA) and then homogenized with glass beads in TissueLyser II (Qiagen, Venlo, Netherlands). RNA was precipitated with TRIzol (Invitrogen, CA, USA) and chloroform and then washed with ethanol to remove any debris of DNA and protein, according to the manufacturer's protocol. The pellet obtained was air-dried and eluted with RNase-free water. The isolated RNA was quantified with Qubit RNA HS Assay kit (Invitrogen), and cDNA was then synthesized in an RT-PCR reaction with *SuperScript Vilo MasterMix* (Invitrogen), using 5 µL RNA and molecular grade water qi 20 µL. The reagents were incubated at room temperature for 10 min, 42 °C for 1 h, and 85 °C for 10 min. We used the Qubit dsDNA HS Assay kit (Invitrogen) to quantify this cDNA and diluted it to 4 ng/µL for qPCR use.

The qPCR reactions were performed in a 96-Well MicroAmp reaction (Invitrogen) plate, with the kit *KAPA SYBR FAST qPCR Master Mix* (1x) and ROX LOW (KAPA Biosystems) kit, 0.2 µM of each forward and reversed primers (Table 5), 4 ng/µL cDNA and molecular grade water *q.s.* 10 µL. Each pool (biological replicate) was divided into four technical replicates. Thermocycling conditions in a QuantStudio 6 Flex Real-Time PCR system (ThermoFisher Scientific) consisted of enzyme activation and initial denaturation at 50 °C and 95 °C for 2 and 3 min, respectively, followed by 40 cycles of denaturation (95 °C for 3 s) and annealing/extension of primers (60 °C for 1 min), with an additional standard melting curve analysis step.

**Table 5:** Primers employed in gene expression analyses.

Gene	Family	Sequences ^a^	Reference
RpS14	Ribosomal protein, housekeeping gene	for: AGGAACTAGCAGAATGGCTCCC rev: ACAGATCCGTGACATGGACGAAG	^94^
CYP6BB2	Cytochrome P450	for: AGTTCAAGGGCCGAGGATTG rev: CGGATCCACGAAAATTCCGC	^58,95,96^
CYP9J10	Cytochrome P450	for: AATACGTACGAGGGATCCAAGA rev: CTATCTCCTCCGACCTCGTCCTC	^29,95^
CYP9J28	Cytochrome P450	for: CTATTTCGGAGTCCTAGTGGCC rev: CTTTGACTCCTCGGTACTTGTCG	^29,58,94–97^
CYP9M6	Cytochrome P450	for: AGCTTGGCAATGACATCATCAC rev: TAAGTCCCTGAAATCCACCAGTG	^29,96^
CCEae3a	Esterase	for: TCTAAGAAACCCGAATATGACG rev: TTGAGGAGGCACGAACAG	^57^
SAP2	Sensory appendage protein	for: TGGAGCCATCAAAGTCATCAA rev: GCGCGATATTGCTCCAGATA	This study

^a^All sequences are in 5′-3′ sense. For and rev indicate forward and reverse, respectively.

#### Analysis of gene expression

We obtained the Ct values of four replicates for each gene and calculated their means. Replicates presenting different Ct from Grubbs values (outliers)^98^ or with an unexpected peak in the melting analysis were excluded. Relative quantification analyses were performed using the ^ΔΔ^Ct method^99^, where the *RpS14* was taken as the reference gene and Rock, reared alongside test population, as the reference strain. For all genes, the Ct threshold was set at 0.2. The 2^(−ΔΔCt)^ equation was applied to define the relative fold-change expression of each gene in PAg compared to Rock. The ΔCt values of each gene were compared between Rock and PAg by unpaired t-test with Welch's correction, performed with GraphPad Prism 8 v 8.4.2 for Mac (GraphPad Software, San Diego, California USA).

## Supplementary Information

Supplementary Information 1.

Supplementary Information 2.
