# Supplementary material for: Insecticide resistance and underlying targets-site and metabolic mechanisms in Aedes aegypti and Aedes albopictus from Lahore, Pakistan
Source: Sci Rep. 2021 Feb 25;11:4555. doi: 10.1038/s41598-021-83465-w (PMC7907206; doi:10.1038/s41598-021-83465-w)
Supplement: Supplementary file 2 — Supplementary Information 2. [file 41598_2021_83465_MOESM2_ESM.pdf]

Insecticide resistance and underlying targets-site and metabolic mechanisms in *Aedes aegypti* and *Aedes albopictus* from Lahore, Pakistan

Rafi Ur Rahman, Barbara Souza, Iftikhar Uddin, Luana Carrara, Luiz Paulo Brito, Monique Melo Costa, Muhammad Asif Mahmood, Sozaina Khan, Jose Bento Pereira Lima, Ademir Jesus Martins

A – *Aedes aegypti*

Aedes aegypti – *Na$_V$* gene - IIS6 segment, exons 20-21

Pakistani population - IIS6 haplotypes

```
                                                                                                                    989
           N  V  D  R  F  P  D  K  D  L  P  R  W  N  F  T  D  F  M  H  S  F  M  I  V  F  R  V  L  C  G  E  W  I  E  S  M  W  D  C [993]
#PAg_2s6_1  ACAATGTGGATCGCTTCCCGGACAAGGACCTGCCACGGTGGAACTTCACCGACTTCATGCACTCATTCATGATCGTGTTCCGGGTATTGTGCGGCGAGTGGATCGAATCCATGTGGGATT [120]
#PAg_2s6_2  ........................................................................................................................

           M  L  V  G  D  V  S  C  I  P  F  F  L  A  T  V  V  I  G  N  L  V                                                    [1015]
#PAg_2s6_1  GTATGCTTGTGGGTGACGTGTCCTGTATTCCGTTCTTTTTGGCCACCGTAGTGATAGGAAATCTAGTAgtaagtattccgtttggaagttcatctgtaaggctgactgaaagtaaattgg [240]
#PAg_2s6_2  ......................................................................................g.........a............g..........

#PAg_2s6_1  agcgcacaacagacctattatgctgtaattcgtgattcaactagttacaaaagaccgttgatcttgatagcatcaatattagaggcgtgctagcagcgagcgaggggcgtaccaatttac [360]
#PAg_2s6_2  ..t...........g....................................t........................c.c.........................................

                                                                    1016
                                                                    V  L  N  L  F  L  A  L  L  L  S  N  F  G  S  S  S  L  S  A [1034]
#PAg_2s6_1  ttttagtcagtctttcttgcattctttcgtgctaaccgacaaattgtttcccactcgcacagGTACTTAACCTTTTCTTAGCCTTGCTTTTGTCCAATTTCGGTTCATCCTCGCTGTCGG [480]
#PAg_2s6_2  ....g....................a............................c.................................................................

           P  T  A  D  N  E  T  N  K  I  A  E  A  F  N  R  I  S  R  F  S  N  W  I  K  S  N  I  A  N  A  L  K  F  V  K  N  K  L  T [1074]
#PAg_2s6_1  CACCGACGGCCGACAACGAAACGAACAAGATCGCGGAGGCGTTCAATCGGATATCGCGCTTCTCCAACTGGATCAAGTCGAACATCGCCAACGCGCTCAAGTTCGTGAAAAACAAGTTAA [600]
#PAg_2s6_2  ........................................................................................................................

           S  Q  I     [1078]
#PAg_2s6_1  CAAGCCAGATTGC [613]
#PAg_2s6_2  .............
```

Underline = primers for sequencing
Squares = common *kdr* sites

Additional information. Supplementary Figure S2.

Insecticide resistance and underlying targets-site and metabolic mechanisms in *Aedes aegypti* and *Aedes albopictus* from Lahore, Pakistan

Rafi Ur Rahman, Barbara Souza, Iftikhar Uddin, Luana Carrara, Luiz Paulo Brito, Monique Melo Costa, Muhammad Asif Mahmood, Sozaina Khan, Jose Bento Pereira Lima, Ademir Jesus Martins

Aedes aegypti – *Na*$_V$ gene – IIIS6 segment
Pakistani population – IIIS6 haplotypes

```
                                                                                                                 1520:T/I
            S  R  E  V  S  Y  C  E  I  E  L  V  T  N  D  L  L  T  I  L  R  P  R  S  F  Q  V  G  K  Q  P  I  R  E  T  N  I  Y  M  Y
#PAg_3s6_1  TCGCGGGAGGTAAGTTATTGTGAAATCGAACTTGTTACGAATGATCTGCTTACAATTTTACGTCCTCGATCCTTCCAGGTGGGAAAGCAGCCGATTCGCGAGACCAACATCTACATGTAC [120]
#PAg_3s6_2  ........................................................................................................................
#PAg_3s6_3  .......................................................................................................T................

                                   1534:F/C
            L  Y  F  V  F  F  I  I  F  G  S  F  F  T  L  N  L  F  I  G  V  I  I  D  N  F  N  E  Q  K  K  K  A  G  G  S  L  E  M  F
#PAg_3s6_1  CTCTACTTTGTGTTCTTCATCATCTTCGGGTCGTTCTTCACGCTGAATCTGTTCATCGGTGTCATCATCGACAACTTCAACGAGCAGAAGAAGAAAGCCGGTGGCTCACTGGAAATGTTC [240]
#PAg_3s6_2  .........................G..............................................................................................
#PAg_3s6_3  .........................G..............................................................................................

            M  T  E  D  Q  K  K  Y  Y  N  A  M  K  K  M  G  S  K  K  P  L  K  A  I  P  R  P  R
#PAg_3s6_1  ATGACGGAGGATCAGAAAAAGTACTACAACGCCATGAAAAAGATGGGCTCGAAGAAGCCGCTGAAAGCTATTCCACGGCCTAGgtaaggcatttccatcgcacatcaac [350]
#PAg_3s6_2  .............................................................................................................
#PAg_3s6_3  .............................................................................................................
```

Similar sequences on GenBank

Haplotype	GenBank accession number
PAg_3s6-1	MF794973, MF794979, MF794985, KM677248, KM677249, KM677255, KM677256, KM677258, KM677249, KY078303, MN413379, KF527415, KY046223, KY046225, KY046228, KY046229, KY046231, KY046237, KP861899, KM519597, MN602782.
PAg_3s6-2	MF794983, MF794989, KM677247, KM677253, KM677278, KM677267, KM677268, KM677266, KM677264, KM677273, KM677276, KM677263, MK005557, MK005556, MK005562, MK005567, MK005563, MK005564, MK005565, MK005566, MK005567, KY078304, MK495873, KF527414, KY046224, KY046232, KY046236, KP861896, MN602780
PAg_3s6-3	MF794977, KM677275, MN602779

Additional information. Supplementary Figure S2.

B – *Aedes albopictus*
Aedes albopictus – Na_V gene – IIS6 segment, exons 20-21
Pakistani population – IIS6 haplotypes

```
                                                                                                 882:L/M
             N  V  D  R  F  P  D  K  D  L  P  R  W  N  F  T  D  F  M  H  S  F  M  I  V  F  R  V  L  C  G  E  W  I  E  S  M  W  D  C
#PAb_2s6-1 ACAATGTGGATCGCTTCCCGGACAAGGACCTGCCGCGGTGGAACTTCACCGACTTCATGCACTCGTTCATGATCGTGTTCCGGGTGCTGTGCGGCGAGTGGATCGAATCCATGTGGGACT   [120]
#PAb_2s6-2 ........................................................................................................................
#PAb_2s6-3 ........................................................................................................................
#PAb_2s6-4 .....................................................................................R..................................
#PAb_2s6-5 ......................................................................................Y.................................

             M  L  V  G  D  V  S  C  I  P  F  F  L  A  T  V  V  I  G  N  L  V
#PAb_2s6-1 GCATGCTGGTCGGCGACGTGTCCTGTATTCCGTTCTTTTTGGCCACCGTAGTGATAGGAAATCTAGTAgtaagtatcctctttcgtccagaagtgctgcgtgaccaacgagtacgtacta   [240]
#PAb_2s6-2 .Y...........................................................................-----....................................ta
#PAb_2s6-3 .........................Y...................................................-------------------------------------------
#PAb_2s6-4 .T...........................................................................-------------------------------------------
#PAb_2s6-5 .Y...........................................................................sy.....k.yymrrmrkksykskkkrn.n.msr.ww.......

                                       V  L  N  L  F  L  A  L  L  L  S  N  F  G  S  S  S  L  S  A  P  T  A  D  N  E  T  N  K  I  A
#PAb_2s6-1 atcggagaatgctttctcccccaatccagGTACTTAACCTTTTCTTAGCCTTGCTTTTGTCCAATTTCGGTTCATCGTCGCTGTCGGCACCGACGGCCGACAACGAAACGAACAAGATCG   [360]
#PAb_2s6-2 ...........y......y.....y...............................................................................................
#PAb_2s6-3 ------------------------------------------------------------------------------------------------------------------------
#PAb_2s6-4 ------------------------------------------------------------------------------------------------------------------------
#PAb_2s6-5 ........................w.y.............................................................................................

             E  A  F  N  R  I  S  R  F  S  N  W  I  K  S  N  I  A  N  A  L  K  F  V  K  N  K  L  T  S  Q  I
#PAb_2s6-1 CGGAGGCGTTCAACCGGATATCGCGCTTCTCCAACTGGATCAAGTCCAACATCGCCAACGCGCTCAAGTTCGTGAAAAACAAGTTAACAAGCCAGATTGC   [460]
#PAb_2s6-2 ....................................................................................................
#PAb_2s6-3 ----------------------------------------------------------------------------------------------------
#PAb_2s6-4 ----------------------------------------------------------------------------------------------------
#PAb_2s6-5 ....................................................................................................
```

Similar sequences on GenBank

Haplotype	GenBank accession number
PAb_2s6-2	MK201618/ MK201609, MK201615, MK201617
PAb_2s6-4	KX281169, KX281170, FJ479615, MK201602/ MK201609, MK201615, MK201617
882 TTG	MF776970

Additional information. Supplementary Figure S2.

Insecticide resistance and underlying targets-site and metabolic mechanisms in *Aedes aegypti* and *Aedes albopictus* from Lahore, Pakistan

Rafi Ur Rahman, Barbara Souza, Iftikhar Uddin, Luana Carrara, Luiz Paulo Brito, Monique Melo Costa, Muhammad Asif Mahmood, Sozaina Khan, Jose Bento Pereira Lima, Ademir Jesus Martins

Aedes albopictus – *Na$_V$* gene – IIIS6 segment
Pakistani population – IIIS6 haplotypes

```
           Q  V  G  K  Q  P  I  R  E  T  N  I  Y  M  Y  L  Y  F  V  F  F  I  I  F  G  S  F  F  T  L  N  L  F  I  G  V  I  D  N
#PAb_3s6-1 CAGGTGGGCAAGCAGCCGATTCGCGAGACCAACATCTACATGTACCTCTACTTTGTGTTCTTCATCATCTTCGGGTCGTTCTTCACCCTCAACCTGTTCATCGGTGTCATCATCGACAAC [120]
#Pab_3s6-2 .....................................................C..................................................................
#Pab_3s6-3 ........................................................................................................................
#Pab_3s6-4 ........................................................................................................................
#Pab_3s6-5 .....................................................Y..................................................................
#Pab_3s6-6 .................R...................................Y...................................Y..Y...........................
#Pab_3s6-7 .................R...................................Y...................................Y..Y...........................
#Pab_3s6-8 .................R...........Y.......................C...................................Y..Y...........................

             F  N  E  Q  K  K  K  A  G  G  S  L  E  M  F  M  T  E  D  Q  K  K  Y  Y  N  A  M  K  K  M  G  S  K  K  P  L  K  A  I  P
#Pab_3s6-1 TTCAACGAGCAGAAGAAGAAAGCCGGTGGCTCGCTGGAAATGTTCATGACGGAGGATCAGAAAAAGTACTACAACGCAATGAAAAAGATGGGCTCGAAGAAGCCGCTGAAAGCTATCCCT [240]
#Pab_3s6-2 ........................................................................................................................
#Pab_3s6-3 ................................A.......................................................................................
#Pab_3s6-4 ................................R.......................................................................................
#Pab_3s6-5 ................................R.......................................................................................
#Pab_3s6-6 ........................................................................................................................
#Pab_3s6-7 ................................R.......................................................................................
#Pab_3s6-8 ........................................................................................................................

             R  P  R  V  R
#Pab_3s6-1 CGGCCTAGGGTAAGG [255]
#Pab_3s6-2 ...............
#Pab_3s6-3 ...............
#Pab_3s6-4 ...............
#Pab_3s6-5 ...............
#Pab_3s6-6 ...............
#Pab_3s6-7 ...............
#Pab_3s6-8 ...............
```

Similar sequences on GenBank

Haplotype	GenBank accession number
PAb_3s6-1	MN433760
PAb_3s6-2	KC1552046, MN433786

Additional information. Supplementary Figure S2.

Insecticide resistance and underlying targets-site and metabolic mechanisms in *Aedes aegypti* and *Aedes albopictus* from Lahore, Pakistan

Rafi Ur Rahman, Barbara Souza, Iftikhar Uddin, Luana Carrara, Luiz Paulo Brito, Monique Melo Costa, Muhammad Asif Mahmood, Sozaina Khan, Jose Bento Pereira Lima, Ademir Jesus Martins

Alignment of IIS6 and IIIS6 segments haplotypes of the voltage gated sodium channel gene of *Aedes aegypti* and Aedes albopictus from Lahore, Pakistan. Nucleotides in the exon and intron regions are in upper- and lowercase letters, respectively. Dots indicate the same nucleotides as the haplotype in the first row. Non-synonymous SNPs changes are in red. The amino acid translation is indicated above the alignment in blue, numbered according to the Na_V protein of *Musca domestica*, as reference. The regions of primers alignment are underlined.
